# A Significance Test for Inferring Affiliation Networks from Spatio-Temporal Data

**DOI:** 10.1371/journal.pone.0132417

**Published:** 2015-07-20

**Authors:** Thomas Furmston, A. Jennifer Morton, Stephen Hailes

**Affiliations:** 1 Department of Computer Science, University College London, London, United Kingdom; 2 Department of Physiology, Development and Neuroscience, University of Cambridge, Cambridge, United Kingdom; Tianjin University, CHINA

## Abstract

Scientists have long been interested in studying social structures within groups of gregarious animals. However, obtaining evidence about interactions between members of a group is difficult. Recent technologies, such as Global Positioning System technology, have made it possible to obtain a vast wealth of animal movement data, but inferring the underlying (latent) social structure of the group from such data remains an important open problem. While intuitively appealing measures of social interaction exist in the literature, they typically lack formal statistical grounding. In this article, we provide a statistical approach to the problem of inferring the social structure of a group from the movement patterns of its members. By constructing an appropriate null model, we are able to construct a significance test to detect meaningful affiliations between members of the group. We demonstrate our method on large-scale real-world data sets of positional data of flocks of Merino sheep, *Ovis aries*.

## Introduction

In recent years, a wide variety of techniques have been developed to obtain animal positional data on a large-scale, and in a relatively cheap manner. This includes video-tracking techniques [1, 2], radio-frequency identification tags [3, 4], such as passive integrated transponder tags [5], and Global Positioning System (GPS) technology [6–8]. The availability of such movement data has led to a surge of research into the use of movement patterns in analysing the social structure of gregarious animals. For example, high-resolution GPS data has been used to study the hierarchical structure of group dynamics in flocks of homing pigeons (*Columba livia domestica*) [6]. Furthermore, video-tracking technology has been used to study the group cohesion in shoals of zebrafish (*Danio rerio*) [2]. The behavioural patterns of gregarious animals are complex and intricate, and the scope for further analyses, as well as the development of new analytical tools, is vast.

Analysing the affiliative or associative structures within a group of animals is a subject that has received much attention. For instance, patterns of associativity that result from the juxtaposition of individual and group type behaviours have been studied in the context of groups of primates [9]. Alternatively, the relation between the availability of food and the social structure of killer whales (*Orcinus ocra*) has been studied [10]. The standard approach to representing the affiliative structure of a group is through an affiliation network, also known as an association network. In such networks, members of the group are represented by nodes. Edges, or links, between animals in the network are typically either binary or weighted. When they are binary, then the presence of an edge is used to indicate that an affiliation exists between the corresponding pair of animals. Otherwise, no such affiliation exists. When the edges are weighted, then the weight of an edge is used to indicate the strength of an affiliation between the two animals. Affiliation networks have a rich representational power, and can provide much information about behavioural patterns within a group of animals. As a result, network-based analysis of animal behaviour has become an increasingly popular family of techniques. (See [11, 12] and references therein for an introduction.)

**Fig 1:**
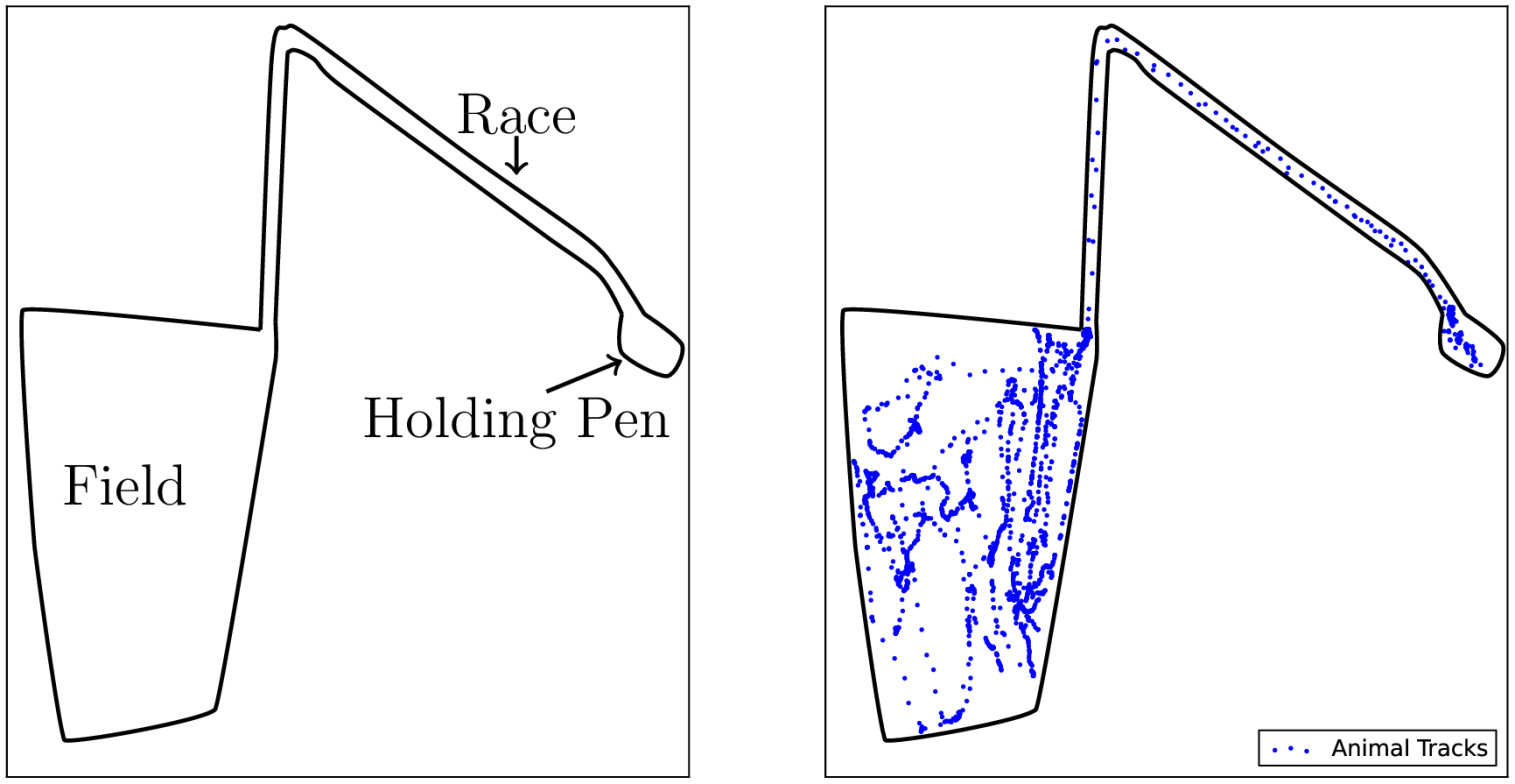
An example of high-resolution positional data collected through GPS technology. This example illustrates the movement patterns of a single sheep from a flock of Merino sheep. The data is split into the period in the holding pen, the period in the race (between the holding pen and the field) and the period in the field. The holding pen and the field are illustrated in the figure on the left, while the tracks of the animal are illustrated (in blue) in the figure on the right. The data set for an entire flock consists of such a trajectory sequence for each individual in the flock. More details of this data set can be found in the data collection section of the paper. Given such a set of movement patterns, the aim of this paper is to provide tools to infer the affiliation network of the group.

This works aims to provide a set of analytical tools that will, given the movement patterns of a group of animals, facilitate the study of affiliative structures within that group. In particular, this paper provides a technique to infer the affiliative network of a group from the movement patterns of its members. Experimental validation of our approach is done using complex real-world high-resolution movement data of Merino sheep, with observations occurring at a rate of 1Hz, that was obtained over the course of hours or days. However, the methods presented in this paper are agnostic to the resolution of the movement patterns, as well as the time-scale of data collection. An illustrative example of the type of data under consideration is given in Fig 1. This example illustrates the movement patterns of a single sheep from a flock of Merino sheep. The data is split into the period in the holding pen, the period in the race (between the holding pen and the field) and the period in the field. The holding pen and the field are illustrated in the figure on the left, while the tracks of the animal are illustrated (in blue) in the figure on the right. The data set for an entire flock consists of such a trajectory sequence for each individual in the flock. Further details of the real-world data sets used to validate the methods presented in this paper are given in the data collection section.

The gambit of the group [13] is the most popular approach in the literature for estimating the affiliation structure of a group of animals. It has been applied in a large range of studies and has proved to be a useful approach for analysing the social structures of animal groups. One difficulty with this approach is that the definition of an affiliation, which forms a central component of the algorithm, is based on either qualitative aspects of the movement data or expert knowledge of the animals under study. As such, the approach lacks any statistical grounding, or any principled approach with which to define an affiliation. Furthermore, the affiliation network obtained from the gambit of the group depends critically on the definition used, which can undermine the validity of the result regarding the social structure of the group. In this paper, we take an alternative approach, in which we consider an affiliation to be present between a pair of animals if there exists significant similarity in their movement patterns. Under this premise, inference about the affiliation network of the group amounts to the determination of significant similarities in the movement patterns its members. We approach this latter problem through the construction of an appropriate significance test. The test uses the observation that, while constructing a movement model for the entire group is a difficult problem, constructing a separate movement model for each individual in the group is straightforward. Using this observation we are able, through the definition of an appropriate null hypothesis, to determine whether any similarity in the movements of two animals is statistically significant, or simply a result of chance. This procedure provides us with a principled procedure to determine the presence of edges in the social structure of the group.

## Gambit of the Group

The gambit of the group [13] is the standard approach for inferring underlying associative structures in a group of animals. The central premise of the gambit of the group is that two animals being in the ‘same place at the same time’ is an indication of an affiliation between two animals. For example, in [14], in which the roosting patterns of brown bats (*Eptesicus fuscus*) are studied, observing two bats in the same roost during the same night is taken an indication of an affiliation between those two bats. Affiliations are noted in this manner for each observation, or window of observations, during the data collection period. This set of observed affiliations is then used to construct an affiliation network for the group. The approach taken to construct the affiliation network depends on various aspects of the study, such as the form of the data collection process. For instance, if not all of the animals are present during each observation, as is often the case in field studies of wild animals, then observational bias [15] needs to be taken into account when constructing an affiliation network. When all the animals are present during each observation, then the standard approach for constructing a weighted affiliation network is to set the edge weights to be the number of observed affiliations (between the corresponding pairs of animals) divided by the total number of observations. This is known as the simple ratio [15]. To obtain a binary network, some form of thresholding is performed, in which an edge is introduced between animals whenever the number of observed affiliations exceeds some threshold.

The validity of the gambit of the group depends on the format of the data, the time-scale over which the data was collected and the aim of the social analysis. It also depends crucially on the definition used to specify an observation of an affiliation. In the case of roosting bats, for example, a roost is a well-defined location and the period of roosting is well understood. When studying roosting patterns, therefore, the definition of an association used in [14] makes clear intuitive sense. Furthermore, given the static nature of roost locations, this approach could easily be formalised through the use of an appropriate significance test to remove spurious links in the network. This would be used much in the manner of [16], in which the foraging patterns of great tits (*Parus major*) were considered. However, in data sets in which such delineations are less apparent, such as in high-resolution movement data, the gambit of the group loses much of its intuitive appeal, and the validity of such definitions becomes more questionable. For instance, in [17], where the social patterns of the guppy fish (*Poecilia reticulata*) are studied, two fish are said to be affiliated if they are within four body lengths of each other during an observation. Likewise, in [7], in which the affiliative structure of Merino sheep was considered, being within two and half metres of each other for three minutes was taken as an indication of affiliation between two sheep. Such definitions are typically based on qualitative, rather than quantitative, aspects of the data. As such, they lack any statistical grounding. Importantly, the absence of any ground truth or test set means that there is no clear method with which to optimise the parameters of such definitions. However, these parameters can have a large impact on the form of the affiliation network generated by the gambit of the group. For instance, in the case of these distance-based definitions, too small a distance will result in important edges being omitted in the network, while too large a distance will result in the introduction of spurious edges. Parameter selection is also an issue in other aspects of the gambit of the group, such as the thresholding parameter that is required for constructing a binary affiliation network. The aim of this paper is to alleviate these issues by taking a statistical approach to the problem of inferring the underlying affiliative structure of a group of animals. In particular, this is done through the introduction of an appropriate significance test.

## Inferring Affiliations from Movement Patterns

In this section we introduce our significance test, which can be used to construct affiliation networks from the movement patterns of a group of interacting animals. The initial emphasis is on the construction of binary affiliation networks; however, we shall later detail several extensions of the significance test to the construction of weighted networks when discussing properties of the significance test. Before constructing the significance test, we first introduce some notation that we will use throughout this section. Given a data set, we use *N* ∈ ℕ to denote the number of animals in the data set and *H* ∈ ℕ to denote the number of time points in the data set. We use the notation (*x*_*t*_(*n*), *y*_*t*_(*n*)) ∈ ℝ^2^ to denote the position of animal *n* ∈ ℕ_*N*_ at time *t* ∈ ℕ_*H*_. (For simplicity we are considering the case where the positional data of each animal is two dimensional, but in general the actual number of dimensions is arbitrary.) We use boldface notation **x**_*t*_, **y**_*t*_ ∈ ℝ^*N*^ to denote the x-coordinate and y-coordinate of the entire group at time *t* ∈ ℕ_*H*_. Finally, we use the notation **x**_1:*H*_, **y**_1:*H*_ ∈ ℝ^*NH*^ to denote the x-coordinates and y-coordinates of the entire group over the entire course of the data collection period. We assume that the positional data of the entire group of animals over the entire course of the data set is contained within a bounded region, and we denote this bounded region by D, and which we shall refer to as the sample space.

### Partition of Sample Space

In order to construct our significance test it is necessary to measure the similarity in the movement patterns of any two animals. To do so, it is necessary to be able calculate the probability that two animals are in the same position at the same point in time. The difficulty here is that D ⊂ ℝ^2^, which means that any probability distribution over D will always put zero probability mass on any two animals being at the exact same position at the same moment in time. To overcome this problem, we partition D into a (finite) collection of subregions, *i.e.* for some *D* ∈ ℕ we construct *D* subregions, {D_*i*_}_*i* ∈ ℕ_*D*__, such that the following conditions are met:

1. The subregions collectively cover the entire data collection area: ⋃_*i* ∈ ℕ_*D*__ D_*i*_ = D.
2. The subregions are disjoint: D_*i*_ ∩ D_*j*_ = ∅ for each *i*, *j* ∈ ℕ_*D*_, such that *i* ≠ *j*.

**Fig 2:**
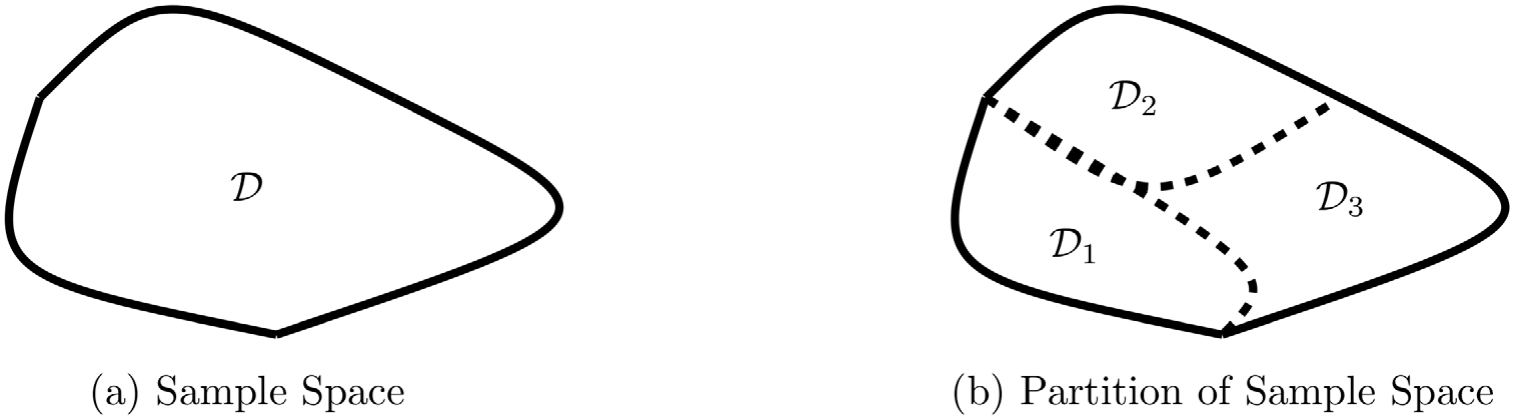
An example partition of the sample space into subregions. The original region is displayed in (a), while a possible partition of this region into three subregions is displayed in (b).

The first condition ensures that each observation is assigned to at least one subregion, while the second condition ensures that each observation is assigned to no more that one subregion. An example of such a partition into subregions is given in Fig 2. The purpose of introducing such a partition is that, given a generative model for the movements of each animal, it is meaningful to calculate terms such as the probability that two animals are in the subregion of the partition at the same point in time. Given such probabilities, we can then determine whether the number of times that two animals were observed to be in the same subregion is significant or simply down to chance. Such a calculation forms the basis of our significance test, but we first detail some possible constructions for the movement models of the individual animals.

### Individual Movement Models

The null hypothesis is that the movement of each animal is independent of the other members of the group. In order to determine whether this null hypothesis should be rejected or not, we need to calculate the probability distribution of each animal’s position over the course of the data set. To calculate these distributions we first learn a movement model for each animal in the group, which we now detail in this section. While constructing a collective movement model for the group is a difficult problem, the construction of a movement model for each individual member of the group is a more straightforward problem, and all that is required under the null hypothesis. There are various possible models that could be considered, but, in this section, we consider two: the first based on a categorical distribution, and the second on a Markov model. To construct such models, we first represent the observations of each animal’s movements in terms of the partition of the sample space. In particular, for each animal, *n* ∈ ℕ_*N*_, and each observation, *t* ∈ ℕ_*H*_, we use the notation *i*_*t*, *n*_ ∈ ℕ_*D*_ to denote the index of the subregion that contains the point (*x*_*t*_(*n*), *y*_*t*_(*n*)), *i.e.* (*x*_*t*_(*n*), *y*_*t*_(*n*)) ∈ D_*i*_*t*, *n*__.

The first model we consider is a categorical distribution. This is a very simple model for which parameter optimisation is straightforward. Denoting the categorical distribution for the *n* ^th^ animal in the group by *π*_*n*_ ∈ ℝ^*D*^, then for any time-point we have

$$\pi_{n}\left(i\right)=\text{probability that animal}\;n\;\text{will be in subregion}\;{𝓓}_{i}.$$

We note that the categorical distribution takes no account of the temporal structure of the data, so, at all time-points, the same probability is given of being in a given subregion. Given the observed movements of animal *n* ∈ ℕ_*N*_ the maximum likelihood estimate of *π*_*n*_, which we denote by ${\hat{\pi }}_{n}$, is given by

$${\hat{\pi }}_{n}\left(i\right)=\frac{C_{i,n}}{\sum_{i^{\prime }\in N_{D}}C_{i^{\prime },n}},$$ (1)

where *C*_*i*, *n*_ is the count of the number of times that animal, *n*, was in subregion, *i*. That is, $C_{i,n}=\sum_{t\in {\mathbb{N}}_{H}}𝕀[i_{t,n},i]$, where $𝕀[i_{t,n},i]$ is the indicator function that is given by

$$I[i_{t,n},i]=\{\begin{array}{cc} 1 & \text{if}\;i_{t,n}=i,\\ 0 & \text{if}\;i_{t,n}\neq i. \end{array}$$ (2)

**Fig 3:**
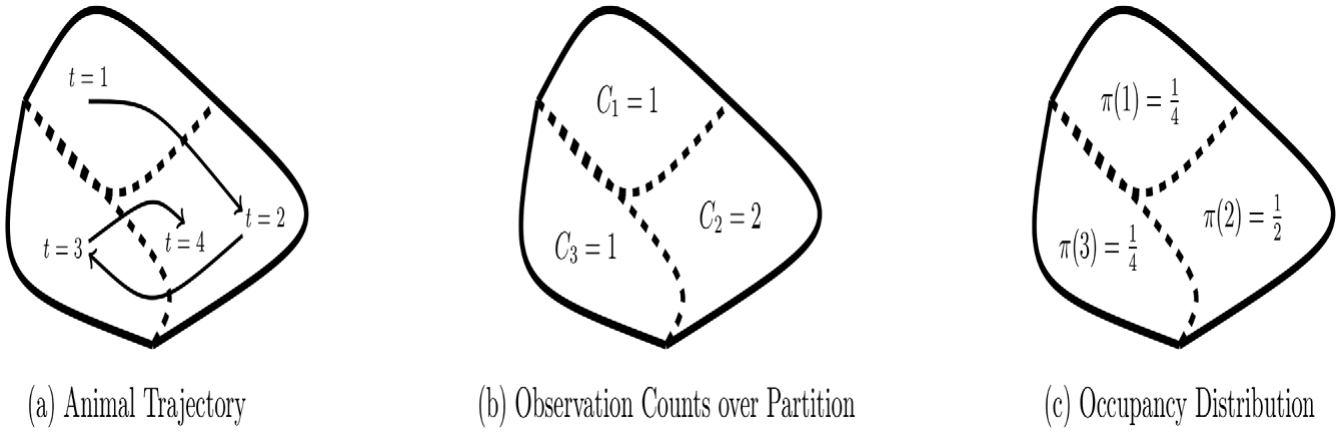
An example of constructing a movement model with respect to a given partition. The partition is the same as given in Fig 2. The animal’s trajectory is given in (a), while the observation counts of the animal into the subregions of the partition is given in (b). Using these observation counts the maximum likelihood estimate of the movement model is given in (c). The animal’s movements have been modelled with a categorical model.

An example of the construction of ${\hat{\pi }}_{n}$ is given in Fig 3.

While a categorical distribution is simple to estimate, it does not take into account the temporal structure of an animal’s movements. For this reason it may be preferable to consider a model that accounts for these temporal dependencies, such as a Markov model. [See *e.g.*[18] for an introduction to Markov models, as well as other time-series models.] We will now consider modelling each animal’s movements through a Markov model, and we construct this model over the partition of the sample space. In a Markov model, we model an animal’s movements by modelling the probability that the animal will be in a given subregion, given that we know the subregion that the animal occupied at the previous point in time. In other words, we construct the conditional distribution, *T*, such that

$$T\left(i|j\right)=\text{probability that the animal will be in subregion}\;{𝓓}_{i}\;\text{given that it was in subregion}\;{𝓓}_{j}\;\text{during the previous time-point}.$$

Constructing a Markov model over the partition of the sample space means that *T* is given by a *D* × *D* matrix, *T* ∈ ℝ^*D* × *D*^, where the *j* ^th^ column of *T* is the probability distribution of the animal’s location given that it was in subregion D during the previous time-point. The model is completed by modelling the position of the animal at the initial time-point. Denoting this distribution by *p* ^0^ ∈ ℝ^*D*^, we have:

$$p^{0}\left(i\right)=\text{probability that the animal will be in subregion}\;{𝓓}_{i}\;\text{during the first time-point}.$$

Given *T* and *p* ^0^, which are referred to respectively as the transition matrix and the initial state distribution, then the movements of the animal are modelled as:

$$p\left(i_{1:H}\right)=p^{0}\left(i_{1}\right)\prod_{t=1}^{H-1}T\left(i_{t+1}|i_{t}\right),$$ (3)

where the occupancy marginal of a given time-point is given by the marginal of *p*(*i*_1:*H*_). When using a Markov model for each individual in a group, we use the notation *T*_*n*_ and $p_{n}^{0}$ respectively to denote the transition matrix and initial state distribution of the *n* ^th^ animal in that group. Given the observations of the *n* ^th^ animal, the maximum likelihood estimate of *T*_*n*_ and $p_{n}^{0}$, which we denote respectively by ${\hat{T}}_{n}$ and $p_{n}^{0}$, are given by:

$${\hat{T}}_{n}\left(i|j\right)=\frac{\sum_{t=2}^{H}I[i_{t,n},i]I[i_{t-1,n},j]}{C_{j,n}-I[i_{H,n},j]},\;\;\;\;\;\;{\hat{p}}_{n}^{0}\left(i\right)=I[i_{1,n},i].$$ (4)

For the example data in Fig 3, the maximum likelihood estimate of the transition matrix and initial state distribution are given by

$$\hat{T}=[\begin{array}{ccc} 0 & 0 & 0\\ 1 & 0 & 1\\ 0 & 1 & 0 \end{array}],\;\;\;\;\;\;{\hat{p}}^{0}=[\begin{array}{c} 1\\ 0\\ 0 \end{array}].$$ (5)

Up to this point we have constructed a separate movement model for each of the individuals in the group. An alternative is to suppose that the movements of the individuals are identically distributed. For instance, when a categorical model is considered, a single categorical model, *π* ∈ ℝ^*D*^, would be used to model the movements of all the individuals in the group. In this case the maximum likelihood estimate of *π*, which is denoted by $\hat{\pi }$, is given by:

$$\hat{\pi }\left(i\right)=\frac{\sum_{n\in N_{N}}C_{i,n}}{\sum_{i^{\prime }\in N_{D}}\sum_{n\in N_{N}}C_{i^{\prime },n}}.$$ (6)

### Determining Significant Coordinated Movements

In this section we detail how the partition of the sample space and the individual movement models can be used to determine whether or not the null hypothesis (of independence between the movement patterns of a pair of animals) should be rejected. The first stage in the test is to determine the number of times that the pair of animals were in the same subregion at the same point in time. For each pair of animals, *n*, *n*′ ∈ ℕ_*N*_, we denote this count by $e_{n,n^{\prime }}^{H}$, so that we have $e_{n,n^{\prime }}^{H}=\sum_{t\in {\mathbb{N}}_{H}}\sum_{i\in {\mathbb{N}}_{D}}𝕀[i_{t,n^{\prime }},i]𝕀[i_{t,n},i]$. We use $e_{n,n^{\prime }}^{H}$ as a measure of similarity between the movements of animals *n* and *n*′, so that higher values of $e_{n,n^{\prime }}^{H}$ correspond to more similar movement patterns. The aim of the significance test is to determine whether this similarity is significant or not. In the second stage of the test, we use the individual movement models to calculate the distribution for the number of co-occurrences between the pair of animals. This distribution can then be used to determine whether the actual number of observed co-occurrences is significant or not. The form of the distribution for the number of co-occurrences depends on the form of the movement model considered, and we now detail the form of this distribution for both the case of a categorical distribution and a Markov model.

When a categorical distribution is used to model the movements of each animal then, for any given pair of animals, *n*, *n*′ ∈ ℕ_*N*_, the probability of a co-occurrence between those animals at a given time point, which we denote by *p*_*n*, *n*′_, is given by:

$$p_{n,n^{\prime }}=\sum_{i\in N_{D}}\pi_{n}\left(i\right)\pi_{n^{\prime }}\left(i\right).$$ (7)

Using the notation $E_{n,n^{\prime }}^{H}$ to denote the random variable for the number of co-occurrences between these two animals over *H* time points, it can be seen that $E_{n,n^{\prime }}^{H}$ follows a binomial distribution with parameters *p*_*n*, *n*′_ and *H*, *i.e.*,

p(En,n′H=e)=(eH)pn,n′e(1-pn,n′)H-e. (8)

This distribution for the number of co-occurrences can now be used to determine whether the observed number of co-occurrences, $e_{n,n^{\prime }}^{H}$, is significant or not. In particular, given a level of significance, *α* ∈ [0, 1], we reject the null hypothesis if

$$p(E_{n,n^{\prime }}^{H}\geq e_{n,n^{\prime }}^{H})\leq \alpha ,$$ (9)

where $p(E_{n,n^{\prime }}^{H}\geq e_{n,n^{\prime }}^{H})$ can be obtained directly from the cumulative distribution for the binomial distribution. If the null hypothesis is rejected we conclude that there is significant interaction between the two animals and so introduce a corresponding edge into the affiliation network.

In the case in which a Markov model is used to model the movements of the animals, the probability of a co-occurrence between two animals depends on the time-point. If, for the pair of animals, *n*, *n*′ ∈ ℕ_*H*_, we use the notation $p_{n,n^{\prime }}^{t}$ to denote the probability of a occurrence between these two animals at time *t*, we have:

$$p_{n,n^{\prime }}^{t}=\sum_{i\in N_{D}}{\hat{p}}_{n}\left(i_{t}=i\right){\hat{p}}_{n^{\prime }}\left(i_{t}=i\right),$$ (10)

where ${\hat{p}}_{n}(i_{t}=i)$ and ${\hat{p}}_{n^{\prime }}(i_{t}=i)$ are the corresponding marginals of Eq (4) under the maximum likelihood estimates of the transition matrix and initial state distribution Eq (5). The distribution for $E_{n,n^{\prime }}^{H}$ no longer has an analytic form, but it can be calculated (in linear time with respect to *H*) in an iterative manner. In particular, for the first time-point we have:

$$p\left(E_{n,n^{\prime }}^{1}=e\right)=\{\begin{array}{cc} 1-p_{n,n^{\prime }}^{1} & \text{if}\;e=0,\\ p_{n,n^{\prime }}^{1} & \text{if}\;e=1, \end{array}$$ (11)

while for all future time-points we have the recursion:

$$p\left(E_{n,n^{\prime }}^{t}=e\right)=\{\begin{array}{cc} \left(1-p_{n,n^{\prime }}^{t}\right)p\left(E_{n,n^{\prime }}^{t-1}=0\right), & \text{if}\;e=0,\\ p_{n,n^{\prime }}^{t}p\left(E_{n,n^{\prime }}^{t-1}=t-1\right), & \text{if}\;e=t,\\ p_{n,n^{\prime }}^{t}p\left(E_{n,n^{\prime }}^{t-1}=e-1\right)+\left(1-p_{n,n^{\prime }}^{t}\right)p\left(E_{n,n^{\prime }}^{t-1}=e\right), & \text{otherwise.} \end{array}$$ (12)

Having used this recursion to obtain the distribution for $E_{n,n^{\prime }}^{H}$ it is then possible to test the significance of the observed number of co-occurrences in the same manner as before.

## Properties of Significance Test

In this section we will discuss some of the properties of the significance test, and how these properties relate to the resulting analyses. Firstly, we discuss several possible ways that the significance test could be used in order to construct weighted networks. We also consider the effect that a heterogeneous environment has on the results of the significance test, as well as the role that the discretisation of the sample space has in the construction of the affiliation network. Finally, we consider the effect that unmarked individuals have on the results of the significance test.

### Construction of Weighted Networks

Thus far we have considered the construction of binary networks of social affiliations. While such networks can contain a wealth of information, it is often desirable instead to construct a weighted network. In weighted networks the weight of each edge is used to indicate the strength of the social affiliation between the corresponding animals. As such, a weighted network of social affiliations not only contains information about the existence of social affiliations, but also the relative importance of those affiliations. In this section we discuss two possible approaches in which the proposed significance test could be used to construct weighted networks.

One approach is to use the information about the number of co-occurrences between animals, $e_{n,n^{\prime }}^{H}$ for *n*, *n*′ ∈ ℕ, to construct the edge weights. Given a pair of animals, *n*, *n*′ ∈ ℕ, denote the critical value of the significance test (at the specified level of significance) between these two animals by *β*_*n*, *n*′_ ∈ ℕ. In other words, the null hypothesis is rejected for any value of $e_{n,n^{\prime }}^{H}$ such that $e_{n,n^{\prime }}^{H}>\beta_{n,n^{\prime }}$. Given the critical value and the number of co-occurrences between the two animals, we calculate the edge weight as follows,

$$w_{n,n^{\prime }}=\{\begin{array}{cc} 0 & \text{if}\;\beta_{n,n^{\prime }}>=e_{n,n^{\prime }}^{H},\\ \frac{e_{n,n^{\prime }}^{H}-\beta_{n,n^{\prime }}}{H-\beta_{n,n^{\prime }}} & \text{otherwise}. \end{array}$$

The significance test is incorporated into the calculation of the edge weight in two ways. Firstly, for those pairs of animals for which there is insufficient evidence to reject the null hypothesis, the edge weight is zero. Secondly, for those pairs of animals for which the null hypothesis is rejected, a baseline is subtracted from the numerator and denominator of the edge weight, with this baseline given by the critical value of the significance test. Subtracting this baseline effectively discounts the number of co-occurrences that can be attributed to chance under the null hypothesis. We refer to this approach as the baseline approach.

Another approach is to first segment the period of data collection into a partition of sub-periods. For instance, a data set consisting of twelve hours of movement data could be separated into four data sets, each consisting of three hours. Given this partition of the original data set, it is then possible to construct a binary network for each of the sub-periods within this partition. These binary networks can then amalgamated to form a weighted network. For instance, by taking the mean of the binary networks.

There are various design choices that would need to be made when considering such an approach, including the construction of an appropriate segmentation of the data set, as well as the training of the movement models. There are numerous approaches that could be taken to obtain a reasonable segmentation of the data. Animal movement patterns often exhibit sudden shifts in the behavioural properties of the subjects, such as those induced by the circadian rhythms of the subjects. For example, gregarious animals tend to wake up and become active at around the same point relative to sunrise. Such shifts in the data could be used to marks points in the segmentation, and could be detected through various unsupervised statistical techniques [18], or through visual inspection of the data. In terms of training the movement models, one possibility is to use the entire data set to train a single movement model, and then to use this model for each of the sub-periods in the partition. Alternatively, if the data set has been partitioned according to different types of activity, then one could train a different movement model for each these different activities.

### Heterogeneous Environments

In many data sets of animal movement patterns the environment is heterogeneous. For instance, it is common for there to be a popular feeding (or watering) location present within the sample space. In such cases, animals will naturally be found more frequently within the vicinity of such locations than in areas that are of less importance to them. Additionally, spatial features of an habitat can have a strong influence on the movement patterns of animals. For instance, the presence of a river can block an animal’s movements, restricting its crossing to a few select places. When using any method to infer the social affiliations of animals from their movement patterns, it is important to understand how the results of that method are affected by any possible heterogeneous aspects of the data.

We now briefly consider the relationship between the results of the significance test and heterogeneous aspects of the animals’ movement patterns. To facilitate our discussion we will consider the example in which a popular feeding location is present within the sample space. The presence of a popular feeding location makes it more likely that any given animal will be found within the vicinity of the feeding location at a given time. Similarly, it also makes it more likely that any two animals will be found within the vicinity of the feeding location at the same time, even when there is no social affiliation between the two animals. For a method to be robust to this heterogeneous aspect of the data, it is necessary that the method can correctly discern whether a large number of co-occurrences is due to a social affiliation between the animals, or simply down to the presence of the feeding location.

**Fig 4:**
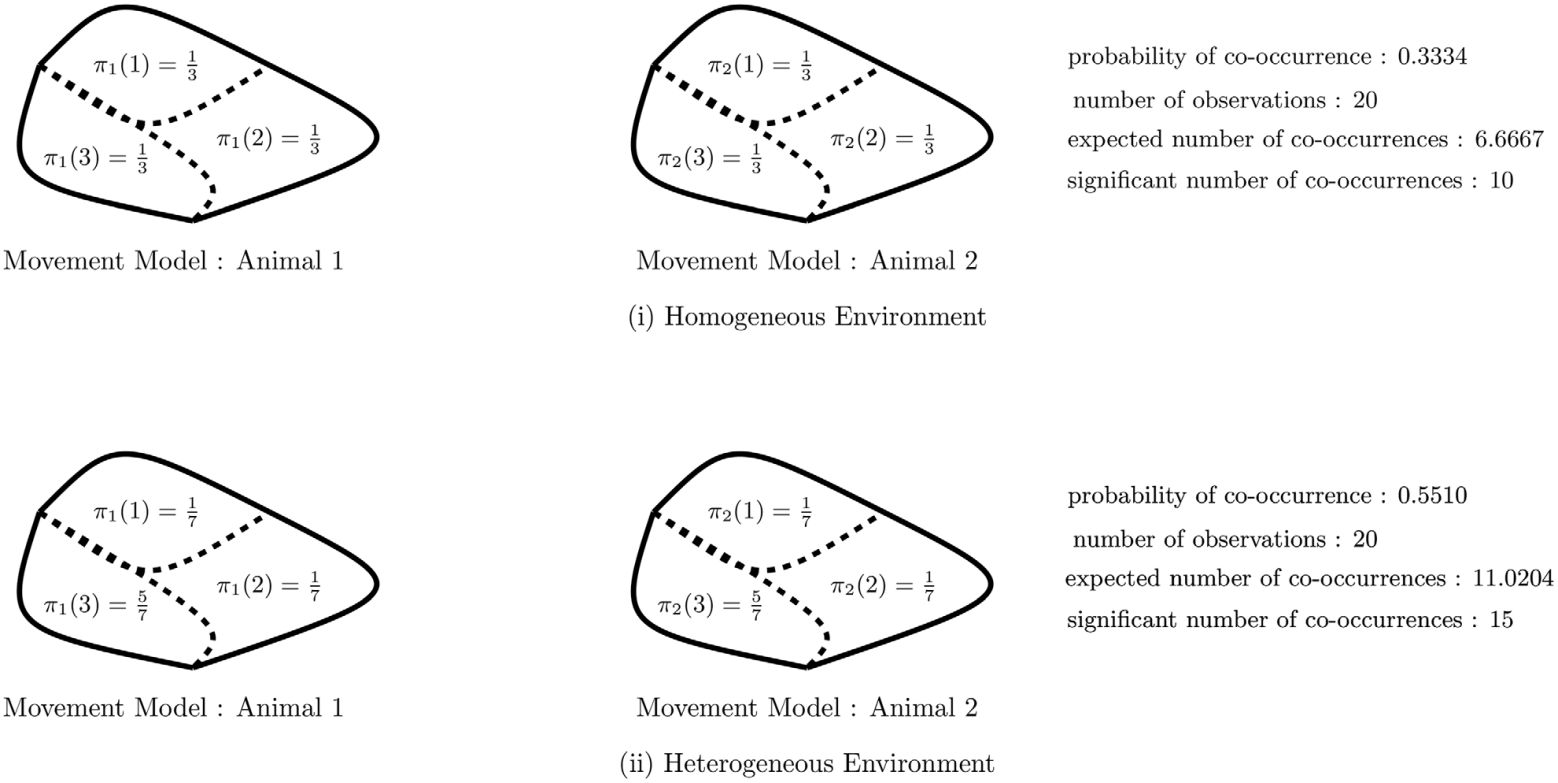
A pictorial example illustrating the effect that heterogeneous data has in the significance test. The top row shows the hypothetical movement models for two animals in an homogeneous environment. There are three bins in the partition of the sample space, and both movement models place equal mass on each bin. The bottom row shows the hypothetical movement models for two animals in an heterogeneous environment. Again, there are three bins in the partition of the sample space. In this environment there is a popular feeding location in the third bin, which means that animals are more likely to spend time in that location. This is shown in the movement models of the two animals. In both examples a categorical model is considered for the movement models of both animals. On the right of both rows is the probability of a co-occurrence, under the null hypothesis of no social affiliation, along with the expected number of co-occurrences and the number of co-occurrences required to reject the null hypothesis (under a 5% level of significance). As can be seen, more co-occurrences are required in the heterogeneous environment to reject the null hypothesis.

The proposed significance test has two important properties of note in relation to the heterogeneous properties of such a data set. The first point is that the movement models of the individual animals, for instance, using a categorical model or a Markov model, will naturally capture the tendency of animals to spend a disproportionately large amount of time within the vicinity of the feeding location. Given that the individual movement models capture this property in the data, it then follows that, under the null hypothesis, there is a higher probability of a co-occurrence at the feeding location. This means that a higher number of co-occurrences are required between two animals in order to obtain a significant result, and to reject the null hypothesis of no social affiliation. It can be seen, therefore, that the significance test is naturally robust to heterogeneous data. A pictorial example of this argument is given in Fig 4.

It is insightful, for the sake of comparison, to also consider the effect that such heterogeneous properties of data can have on the results given by gambit of the group. Under the gambit of the group, a social affiliation would be noted between two animals whenever those two animals happen to be present at the feeding location at the same time. A consequence of this is that the gambit of the group would indicate more social affiliations than are actually present in the data, giving misleading results.

### Discretisation of Sample Space

Constructing a finite partition of the sample space forms an important point in our significance test. If the partition is too fine, then it may lead to poor estimates of the movement model. If the partition is too coarse, then the movement model may be insufficiently rich to discern independence accurately, or the lack thereof. Our use of a finite partition is analogous to other independence tests in the statistics and machine learning literature, such as those based on the *L*_1_-divergence measure [19], on the log-likelihood [19] and on contingency tables [20]. (We note that these approaches assume that the data are independent and identically distributed, which is not the case in our problem setting, where the data have a temporal structure.) Consistent methods have been constructed for both the *L*_1_-divergence measure and the log-likelihood, in which the partition becomes more refined as the sample size increases. See [19] for more details. It is a matter of future work to to provide such consistency results for our framework.

We will now briefly discuss several practical approaches to constructing the partition, which in part are related to our discussion of heterogeneous environments. A simple approach to constructing the partition of the sample space is to split the sample space into *N* regularly shaped cells of the same shape. In a two-dimensional sample space, for example, the sample space would first be bounded by a rectangular cell, and this rectangular cell would then be split into *N* rectangles of the same size. An alternative approach that is common in similar types of significance test is to construct an irregular partition of the sample space in such a manner that (roughly) the same amount of data falls into each of the cells of the partition. This point is also related to the earlier discussion of heterogeneous environments. Again, consider the example in which a popular feeding location is present within the sample space. Due to the popularity of the feeding location, much of the data would be observed within the relatively small part of the sample space. A regular discretisation of the sample space could lead to poor results in this case, perhaps with much of the data falling into a single cell that contains the feeding location. Instead, an irregular discretisation could be more appropriate, with a fine discretisation in and around the feeding location, and a coarse discretisation in other areas of the sample space. In this manner, an irregular discretisation could help improve the results of the significance test, focusing the representational power of the movement model into important areas of the sample space. An algorithm to discretise the sample space into irregular grids, with approximately the same number of observations in each grid, is given in algorithm 1 in Table 1.

**Table 1:** An Algorithm to Discretise Sample Space into Irregular Rectangles.

**Inputs:**
Observations of each animal’s position during the data collection period, ${\left\{\left(x_{t}(n),y_{t}(n)\right)\right\}}_{n=1,}^{N}{}_{t=1}^{H}$, in which (*x*_*t*_(*n*), *y*_*t*_(*n*)) ∈ ℝ^2^.
A rectangular region that contains the sample space, $𝓓=\left[x^{\text{low}},x^{\text{high}}\right]\times \left[y^{\text{low}},y^{\text{high}}\right]\in R^{2}$ such that (*x*_*t*_(*n*), *y*_*t*_(*n*)) ∈ 𝓓 for all *n* = 1, .., *N* and *t* = 1, …, *H*.
The desired number of cells in the partition, *D* ∈ ℕ.
**Algorithm:**
Initialise partition: set 𝓓_1_ = 𝓓.
Calculate observation counts of subcells: *C*_j_ = *N* × *H*.
**for** i = 1, 2, …., D-1 **do**
Find subcell with largest number of observations, ${𝓓}_{i*}\in {\left\{{𝓓}_{j}\right\}}_{j=1}^{i}$ such that $i^{*}={\mathrm{argmax}}_{j\in N_{i}}\;C_{j}$.
Split 𝓓_i*_ into two subcells such that half the observations in 𝓓_i*_ fall into each of the two subcells.
For instance,
**if** $x_{i^{*}}^{\text{high}}-x_{i^{*}}^{\text{low}}\geq y_{i^{*}}^{\text{high}}-y_{i^{*}}^{\text{low}}$, **then**
find $x_{i^{*}}^{\text{middle}}\in \left(x_{i^{*}}^{\text{low}},x_{i^{*}}^{\text{high}}\right)$ such that the subcells
$${𝓓}_{1}^{\text{new}}=\left[x_{i^{*}}^{\text{low}},x_{i^{*}}^{\text{middle}}\right]\times \left[y_{i^{*}}^{\text{low}},y_{i^{*}}^{\text{high}}\right],$$
$${𝓓}_{2}^{\text{new}}=\left[x_{i^{*}}^{\text{middle}},x_{i^{*}}^{\text{high}}\right]\times \left[y_{i^{*}}^{\text{low}},y_{i^{*}}^{\text{high}}\right],$$
such that ${𝓓}_{1}^{\text{new}}$ and ${𝓓}_{2}^{\text{new}}$ contain the same number of observations. This can be done, for example, through the bisection method.
**else**
find $y_{i^{*}}^{\text{middle}}\in \left[y_{i^{*}}^{\text{low}},y_{i^{*}}^{\text{high}}\right]$ such that the subcells
$${𝓓}_{1}^{\text{new}}=\left[x_{i^{*}}^{\text{low}},x_{i^{*}}^{\text{high}}\right]\times \left[y_{i^{*}}^{\text{low}},y_{i^{*}}^{\text{middle}}\right],$$
$${𝓓}_{2}^{\text{new}}=\left[x_{i^{*}}^{\text{low}},x_{i^{*}}^{\text{high}}\right]\times \left[y_{i^{*}}^{\text{middle}},y_{i^{*}}^{\text{high}}\right],$$
such that ${𝓓}_{1}^{\text{new}}$ and ${𝓓}_{2}^{\text{new}}$ contain the same number of observations. This can be done, for example, through the bisection method.
**end if**
Update partition: remove 𝓓_i*_ from partition, and introduce ${𝓓}_{1}^{\text{new}}$ and ${𝓓}_{2}^{\text{new}}$ into partition.
Update indices of subcells in the partition.
Calculate observation counts of subcells: *C* = # observations in 𝓓_*j*_, for *j* = 1, 2, …, *i* + 1.
**end for**

### Presence of Unmarked Individuals

It is often the case that animals were present in the sample space for which no movement data is available. For instance, if the group under observation contained a large number of animals, then it may not have been practical to log the movements of the entire group. Alternatively, logging equipment can fail during the course of the data collection process, resulting in data being lost for some members of the group. It is important, therefore, to understand the effect that unmarked individuals have on the results of the significance test. To do so, consider the hypothetical case in which there are two marked animals and one unmarked animal. There are several possible situations to consider. If the two marked animals have a direct affiliation, then the significance test will typically reject the null hypothesis, regardless of whether there is any affiliation with the unmarked animal or not. Assume, therefore, that there is no direct affiliation between the two marked animals. If there is no direct affiliation between the unmarked animal and either of the marked animals, or at most one of the marked animals, then there is no reason to expect that the significance test will reject the null hypothesis and indicate a social affiliation between the two marked animals. The remaining case is that there is a direct affiliation between the unmarked animal and both of the marked animals, but no direct affiliation between the two marked animals. In such a situation the two marked animals would tend to be seen in the same vicinity as each other, even though their social affiliation is only of a transitive nature though the unmarked individual. As a result the significance test would typically reject the null hypothesis and conclude that the two marked individuals have a social affiliation. Whether such a result is reasonable (in terms of the analysis to be performed on the resulting network) depends on the form of the experiment being undertaken, and is a point that must be considered as necessary.

## Data Collection

We demonstrate the significance test on large-scale real-world data sets of positional data of flocks of Merino sheep, *Ovis aries*. The data were collected as part of a project aimed at measuring flock behaviour in naturalistic settings. The GPS data were collected via GPS loggers of an in-house design, a detailed description of which can be found in [7]. The GPS data were post-processed using the open-source RTKLib and GeographicLib libraries respectively to give Cartesian coordinates with respect to a local projection. (These libraries are freely available at http://www.rtklib.com/ and http://geographiclib.sourceforge.net/.) Given the post-processed data, the geographical positional data of each animal were smoothed using a linear dynamical system [18] for which the parameters were optimised using the Expectation-Maximisation algorithm [21]. This removed much of the noise, which was mainly Gaussian in nature, and provided estimates of any observations that were missing due to failures in signal acquisition. The data sets were roughly 24 hours in length, but this varied according to various factors, such as the battery life of the logging equipment. Each data set consists of an initial period during which the loggers were attached to each animal (whilst in a holding pen); a period in which all the animals were herded down a race into their field; a middle period where the animals were left to roam in the field; and then a final period during which the animals were herded back into the holding pen to have logging equipment removed. A visual illustration of the data for a single sheep is given in Fig 1. In all of the experiments we focus on the sections of data in which the flock is contained within the field, which is when they are exhibiting their natural behaviour.

In view of the fact that sheep are slow-moving animals, we decided to collect GPS data at a rate of 1 sample/s. This rate was chosen empirically, as we considered it would be more than sufficient to capture interesting behavioural aspects in the movement patterns of the animals in the study. This assumption was validated by analysing data from the genuine mixing experiment, that was reprocessed to give different capture rates.

Using the original data set collected, across all sheep, average velocity was determined to be 0.050516 m/s (95% CI [0.0498 0.0512]). To assess whether a higher sampling rate would have added significantly to the accuracy of the positional estimates, we reprocessed the data, examining the effect of removing varying numbers of samples, then estimating their values and comparing the quality of that estimate with the original data. The procedure used was as follows:

1. Take the raw, unfiltered data for each logger
2. Subsample it so that only a proportion of the data are marked as valid
3. Linearly interpolate to generate estimates for the invalid values. Extrapolate at the start and end simply by repeating the first/last valid value.
4. Compare the regenerated time series against all the valid values in the original.

This approach uses an unsophisticated estimator and operates on the raw data without any attempt to remove GPS artefacts, so is likely to provide a strong indication of the likely errors in sampling more slowly, and, by extension, the benefits of sampling faster. The results are given in Table 2.

**Table 2:** The positional error of linearly interpolated data. The table shows the results of reducing sampling rate from 1 sample/s, linearly interpolating to reconstruct the missing values, and comparing against the original data. The results support the assumption that a GPS data sampling rate of 1 sample/s is likely to be more than adequate as a means of establishing sheep positions for this study, and that little would be gained by increasing it.

Sampling rate sample/s	% Original data remaining	Mean distance error /m	95% CI
1	100%	0	0
0.5	50%	0.0104	[0.0101 0.0106]
0.25	25%	0.0266	[0.0261 0.0270]
0.125	12.5%	0.0586	[0.0579 0.0594]
0.06125	6.125%	0.1258	[0.1243 0.1274]
0.030625	3.0625%	0.2547	[0.2518 0.2576]

As can be seen, halving the sampling rate adds a mean distance error of around 1cm. Even if we remove 97% of the original data, we add an average positional error of only ~25cm. This would suggest that linear interpolation works well in terms of estimating position for missing values, even between temporally distant samples. So, if one considers the potential benefits of obtaining data at 10 samples/s (the maximum sampling rate of the equipment), instead of the 1 sample/s we chose, the gain in measuring at the higher rate would be on the order of millimetres at best. This would have no impact on the results of the study. In short, as expected, a sampling rate of 1 sample/s is significantly higher than is necessary for the behavioural analysis of these slow moving animals.

## Experiments

We validate the significance test using the real-world positional data of Merino sheep. Validation is a non-trivial problem because the ground truth (*i.e.* the actual underlying affiliation network of the group) is not available. We therefore construct three experiments that allow us to determine the ability of the significance test to represent the ground truth accurately; a diseased sheep experiment, an artificially-generated mixing experiment, and a genuine mixing experiment. In the diseased sheep experiment, the details of which are given below, the flock is formed of a collection of isolated nodes, with each isolated node corresponding to one of the diseased animals, and a connected sub-flock, consisting of the sheep in the flock that are not diseased. In the two mixing experiments, the details of which are given below, the flock is formed of two sub-flocks, and interaction within each of these two sub-flocks is higher than it is between them. We use this a-priori knowledge of the flock structure to determine the ability of the significance test in inferring the affiliation network of the flock. The data used in the experiments, along with the code used to perform the experiments, is freely available from https://www.repository.cam.ac.uk/handle/1810/247648. Additionally, we have written a Matlab [22] toolbox of the methods presented in this paper. The toolbox is freely available from the same location.

### Batten Disease Sheep Experiment

In the Batten disease sheep experiment, we consider a data set that consists of eleven individual sheep, recorded over the course of six days. Five of these individuals carried a mutation in *CLN5* that causes a progressive neurological disease that has a significant impact on their behaviour [23, 24]. The remaining six sheep did not suffer from the disease and were healthy. The affected animals were at an advanced stage of the disease and their interaction with other animals was negligible. As such, it is to be expected that the affiliation network of these eleven animals will consist of a sub-flock, composed of the six normal animals, along with five isolated nodes, i.e. nodes with no edges, with one such node for each of the diseased animals.

**Fig 5:**
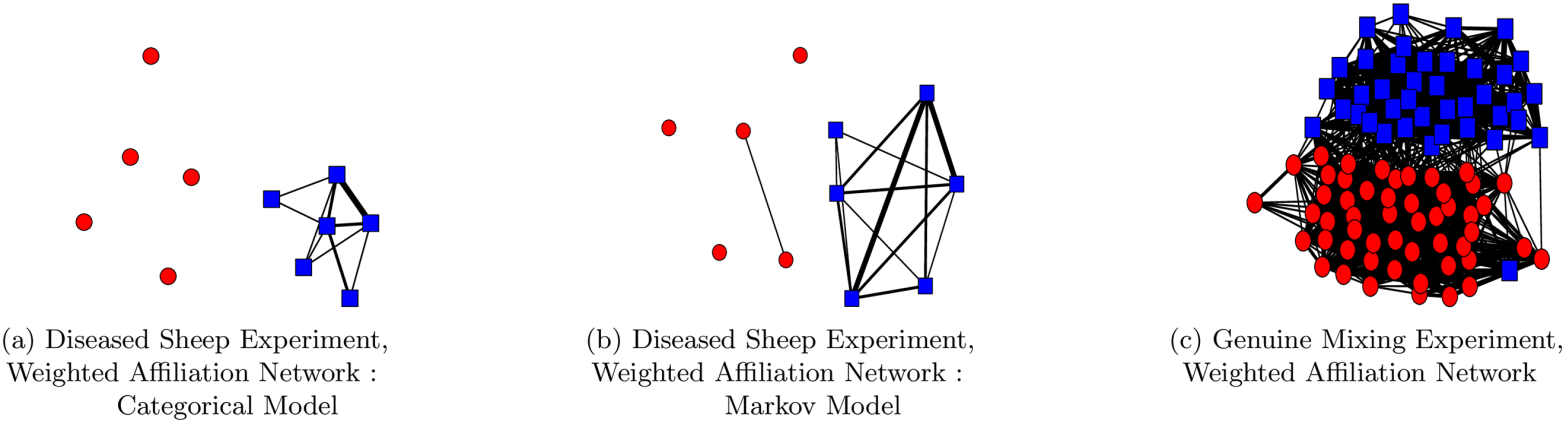
Weighted affiliation networks generated using the significance test. (a-b) Weighted affiliation networks generated for the diseased sheep data set using the significance test. The network in (a) was constructed using a categorical model, while the network in (b) was constructed using a Markov model. Red circular nodes are used to represent diseased animals, while blue square nodes are used to represent healthy animals. (c) Weighted affiliation network generated for the genuine mixing experiment using the significance test. Red circular nodes are used to represent members of one of the pre-mixed flocks, while members of the other pre-mixed flock are represented using blue square nodes. In all graphs edges with a weight less than 0.1 have been removed as these edge weights were considered negligible.

We construct a separate weighted network for each of the six different days. For each of the days we consider a six hour period, selecting periods during which there is a high amount of movement activity and during which the flock are within the field. For each of the days the sample space is partitioned by first finding a rectangular region that bounds the field. This rectangular area is then split into twenty five equally sized subregions. We use a 0.5% level of significance in the significance test, and use the baseline approach to calculate the edge weights. We take the mean of the six weighted networks to obtain a final single weighted network for the flock. We consider both the categorical model and the Markov model. In both cases we use the data of the entire group to construct a single model. In the Markov model we take the median position of each individual over a five minute period as an observation. In the categorical model we take the median position of each individual over a fifteen minute period as an observation. We consider a longer period for an observation in categorical model in order to reduce the auto-correlation in the observations. The weighted affiliation networks for the categorical model and the Markov model are given in Fig 5, and directly corroborate the hypothesised affiliative structure of this group of animals.

**Fig 6:**
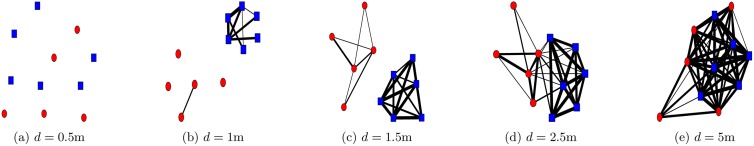
Weighted affiliation networks generated using the gambit of the group. Weighted affiliation networks generated using the gambit of the group for the diseased sheep data set. All five networks (a-e) were constructed using exactly the same data set, and differ only in the value of the parameter, *d* ∈ ℝ^+^, used to define an association in the gambit of the group. The value used to generate each network is given in the caption of the corresponding network. Red circular nodes are used to represent diseased animals, while blue square nodes are used to represent healthy animals. In all graphs edges with a weight less than 0.1 have been removed as these edge weights were considered negligible.

To demonstrate the advantages that our statistical approach has in comparison to the gambit of the group, we also apply the gambit of the group to the diseased sheep data set. In order to apply the gambit of the group to this data set it is necessary to construct a definition for an association between two animals. We use the definition given in [7], which also considered Merino sheep. In this definition, an affiliation is said to be present between two animals if they are within *d* metres of each for at least *β*% of the observation window. As such, there are three parameters that need to be set in this definition, *d* ∈ R^+^ and *β* ∈ [0, 100], along with the length of the observation window. We use three minute observation windows and set *β* = 5. (Qualitatively similar behaviour was obtained for different lengths of observation window and different values of *β*. The length of observation window used was found to be ‘optimal’ according to the K-means clustering experiment in [7].) We consider different values of *d* and examine the sensitivity of the affiliation network with respect to this parameter. As in the case of the significance test, we use the gambit of the group to generate weighted affiliation networks. All animals were seen during each observation window, so the simple ratio [15] was used to calculate the edge weights. We consider five different values of *d*, with *d* = 0.5m, *d* = 1m, *d* = 1.5m, *d* = 2.5m and *d* = 5m. The resulting affiliation networks are given in Fig 6. The sensitivity of the affiliation network with respect to the value of *d* can be seen from the different networks in Fig 6. For the setting, *d* = 0.5m, the affiliation network is completely disconnected, suggesting that there are no affiliations between any of these animals. Given the gregarious nature of the healthy members of the flock, it is highly likely that the healthy animals form a connected sub-flock in the true affiliation network. The affiliation network that bears the most resemblance to those generated by the significance test, and those expected both through our understanding of the diseased animals and through a visual inspection of the data, is that given by the setting, *d* = 1m. The results for both *d* = 1.5m and *d* = 2.5m suggest the presence of two sub-flocks, one consisting entirely of diseased animals and the other consisting entirely of healthy animals. In the case of *d* = 1.5m, the results indicate no association between these two sub-flocks, while in the case of *d* = 2.5m, the results indicate that there is association between these two sub-flocks, but that it is weaker than the association within the two sub-flocks. Expert knowledge of these animals, obtained through visual observation of the behaviour of the diseased animals during the field trip, gives an explanation for these results. In particular, it was noted that when any of the healthy animals happened to move past a diseased animal, then, on occasion, the diseased animal would follow the healthy animals for a brief amount of time. As such, there were occasions when several of the diseased animals would happen to follow the same healthy animals at the same time, even though there was no affiliative behaviour between the diseased animals themselves. The gambit of the group is identifying this aspect of the data when a value of *d* = 1.5m or *d* = 2.5m is used. It is only through this expert knowledge of the data, however, that it is possible to explain this aspect of these results. Without this expert knowledge one could infer, incorrectly, a dichotomous flock structure that is not present in this group of animals. It is worth noting that the setting *d* = 2.5m was found to be ‘optimal’ for Merino sheep in [7], so that this value of *d* is not extreme in terms of values that have been used previously in the literature. For the setting, *d* = 5m, the affiliation network is highly connected and all but one of the diseased animals appear to be exhibiting strong affiliations with the healthy animals. These results illustrate that the affiliation network generated through the gambit of the group is sensitive with respect to the parameters used to define an association, with markedly different results were obtained through small changes to these parameters. This sensitivity, along with the lack of any principled approach to setting these parameters, is a highly undesirable aspect of this approach.

We now consider the sensitivity of the results of the significance test in terms of the size of the observation window and the number of subregions in the discretisation of the sample space. In particular, we apply the significance test to the Batten disease sheep data in the same manner as before, but now consider different lengths of observation window and different number of subregions in the discretisation of the sample space. In this analysis we consider a Markov model for the individual movement models. We consider observation windows of ten different lengths, ranging from one minute to ten minutes in length. The sample space is two-dimensional, and we consider splitting each dimension into 3, 4, 5, 6, 7, 8, 9 & 10 equally sized bins. The results of the experiment are given in Table 3. It can be seen that the method is robust to both the length of the observation window and the number of subregions in the discretisation. In all cases the methods is able to correctly identify that the healthy animals form a connected component. Additionally, the mean weight of edges between healthy animals is consistently higher than the mean weight of edges which include a diseased animal as one of its vertices. It can be seen that the mean weight (both for edges between healthy animals and for edges involving a diseased animal) decreases as the number of subregions in the discretisation increases. This is as expected. Indeed, the finer the discretisation of the sample space the lower the likelihood that two animals will be within the same subregion at the same point in time. As mentioned previously, the estimates of the movement model will become poor as the discretisation becomes too fine. This is not apparent for the broad range of discretisations that we considered, which indicates an acceptable amount of robustness in this aspect of the significance test. Finally, the number of edges involving a diseased animal that are absent from the affiliation network increases with the length of the observation window. The Markov model relies on the Markov assumption, i.e. that the location of an animal at one time point depends only on the location of the animal at the previous time point. There is a relation between the length of the observation window and the validity of the Markov assumption, with the validity of the Markov assumption decreasing as the length of the observation window decreases. This leads to a higher false-positive rate for shorter observation windows, and thus the lower number of omissions of edges involving a diseased animal. Conversely, to obtain a sufficient number observations to reliably fit a movement model it is necessary to consider observation windows that are not too long in length. This interplay between the length of the observation window and the assumptions made in the individual movement models is important. The results suggest, however, that in practice our approach is reasonably robust in this regard.

**Table 3:** Results from the sensitivity analysis of the significance test. The table shows the results in terms of the size of observation window and the number of subregions in the partition of the sample space. The sample space is two-dimensional, and the discretisation is given in terms of the split across each dimension. The results give (i) the number of absent edges for which at least one vertex is a diseased animal (ii) the mean edge weight of those edges for which at least one vertex is a diseased animal (iii) the size of the largest component of healthy animals (iv) the mean edges weight between healthy animals.

		Size of Discretisation							
Observation Window		3 × 3	4 × 4	5 × 5	6 × 6	7 × 7	8 × 8	9 × 9	10 × 10
60 Seconds	# of absent diseased edges	6	3	3	3	2	7	6	6
	mean diseased edge weight	0.08	0.05	0.04	0.03	0.03	0.03	0.03	0.02
	size of healthy component	6	6	6	6	6	6	6	6
	mean inter-healthy edge weight	0.30	0.25	0.24	0.20	0.18	0.18	0.16	0.15
120 Seconds	# of absent diseased edges	7	3	3	10	5	11	9	8
	mean diseased edge weight	0.07	0.05	0.04	0.03	0.03	0.03	0.02	0.02
	size of healthy component	6	6	6	6	6	6	6	6
	mean inter-healthy edge weight	0.29	0.25	0.23	0.19	0.17	0.18	0.14	0.14
180 Seconds	# of absent diseased edges	11	12	9	11	7	10	10	15
	mean diseased edge weight	0.07	0.05	0.04	0.03	0.02	0.02	0.02	0.02
	size of healthy component	6	6	6	6	6	6	6	6
	mean inter-healthy edge weight	0.27	0.23	0.22	0.18	0.16	0.16	0.14	0.13
240 Seconds	# of absent diseased edges	14	14	10	14	7	17	9	19
	mean diseased edge weight	0.07	0.05	0.03	0.02	0.02	0.02	0.02	0.02
	size of healthy component	6	6	6	6	6	6	6	6
	mean inter-healthy edge weight	0.25	0.22	0.20	0.17	0.15	0.15	0.12	0.13
300 Seconds	# of absent diseased edges	15	16	15	15	10	16	12	21
	mean diseased edge weight	0.07	0.05	0.04	0.03	0.02	0.02	0.02	0.02
	size of healthy component	6	6	6	6	6	6	6	6
	mean inter-healthy edge weight	0.24	0.21	0.20	0.16	0.14	0.15	0.13	0.12
360 Seconds	# of absent diseased edges	20	17	19	18	18	20	19	17
	mean diseased edge weight	0.07	0.05	0.04	0.03	0.02	0.02	0.02	0.02
	size of healthy component	6	6	6	6	6	6	6	6
	mean inter-healthy edge weight	0.21	0.21	0.20	0.15	0.14	0.14	0.12	0.11
420 Seconds	# of absent diseased edges	21	19	22	19	21	23	22	20
	mean diseased edge weight	0.06	0.05	0.04	0.03	0.02	0.03	0.02	0.02
	size of healthy component	6	6	6	6	6	6	6	6
	mean inter-healthy edge weight	0.22	0.21	0.19	0.16	0.13	0.14	0.11	0.12
480 Seconds	# of absent diseased edges	18	18	22	27	21	22	23	32
	mean diseased edge weight	0.06	0.04	0.03	0.03	0.02	0.02	0.02	0.02
	size of healthy component	6	6	6	6	6	6	6	6
	mean inter-healthy edge weight	0.19	0.19	0.18	0.13	0.12	0.13	0.10	0.10
540 Seconds	# of absent diseased edges	23	21	25	26	30	25	31	28
	mean diseased edge weight	0.06	0.04	0.04	0.03	0.02	0.02	0.03	0.02
	size of healthy component	6	6	6	6	6	6	6	6
	mean inter-healthy edge weight	0.19	0.16	0.16	0.15	0.11	0.12	0.09	0.10
600 Seconds	# of absent diseased edges	23	24	29	31	33	27	30	29
	mean diseased edge weight	0.06	0.05	0.04	0.03	0.03	0.02	0.02	0.02
	size of healthy component	6	6	6	6	6	6	6	6
	mean inter-healthy edge weight	0.16	0.18	0.16	0.12	0.11	0.12	0.08	0.09

### Artificial Mixing Experiment

In the artificial mixing experiment, we posit that there should be little to no correlation in the movement patterns of the animals over two different days. Using this supposition it is possible to construct artificial data sets that exhibit the structure of a flock that is clearly separated into two sub-flocks. In particular, we take pairs of individual data sets and amalgamate them into a single data set. To ensure that there is a clear demarcation between the two sub-flocks, we amalgamate data sets from different days, e.g., the 1^st^ and 2^nd^ of March. We only consider pairs of data sets that were collected in the same field. This is done to ensure that the overlap (of the movement patterns) of the two sub-flocks is substantial, and the experiment is appropriately challenging. This results in a total of twenty three different amalgamated data sets, with an average of one hundred and forty animals in each.

To partition the sample space we first find a rectangular region that bounds the field. This rectangular area is then split into twenty five equally sized subregions. We only consider periods during which the group are within the field and, where necessary, we truncate the data to ensure that the individual data sets are of the same length. We consider a Markov model, and use the data of the entire group to construct a single model. We take the median position of each individual over a five minute period as an observation. We use the significance test to construct a single binary network, and consider a 0.5% level of significance.

**Fig 7:**
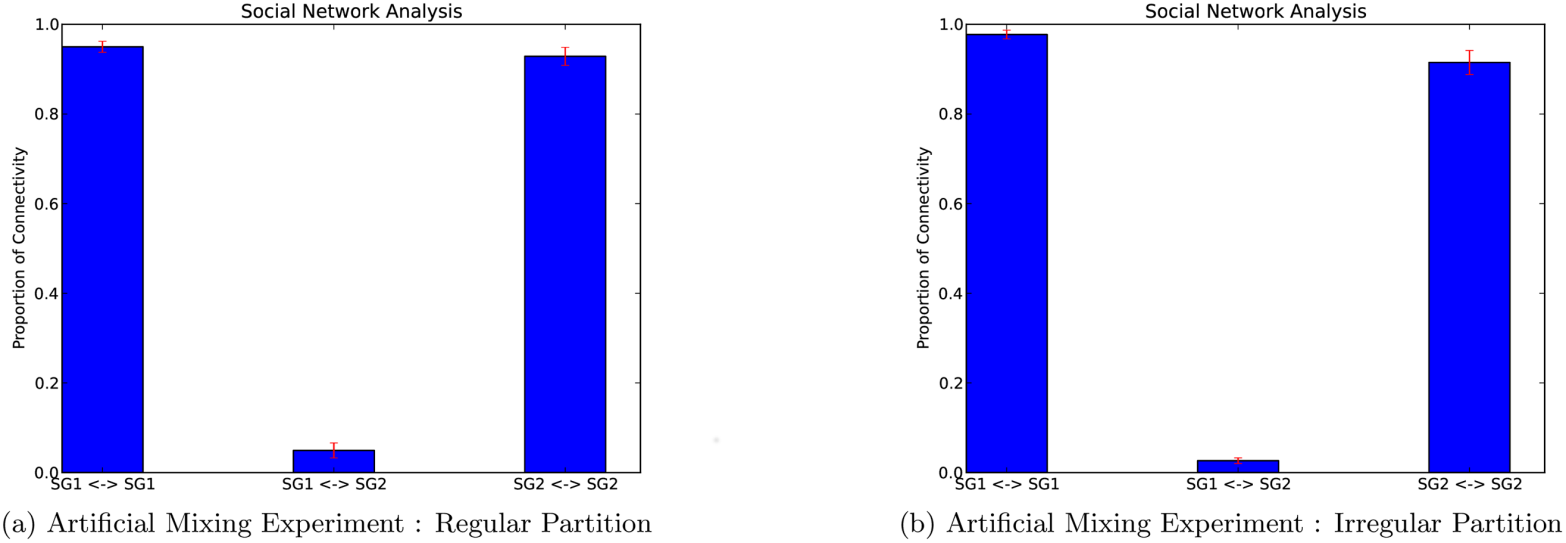
Results of the artificial mixing experiment. The plot shows the proportion of connections within the two sub-flocks, *SG*1 ↔ *SG*1 and *SG*2 ↔ *SG*2, and between the two sub-flocks *SG*1 ↔ *SG*2. The plots show the results of the significance test using a regular partition (a) and an irregular partition (b).

**Fig 8:**
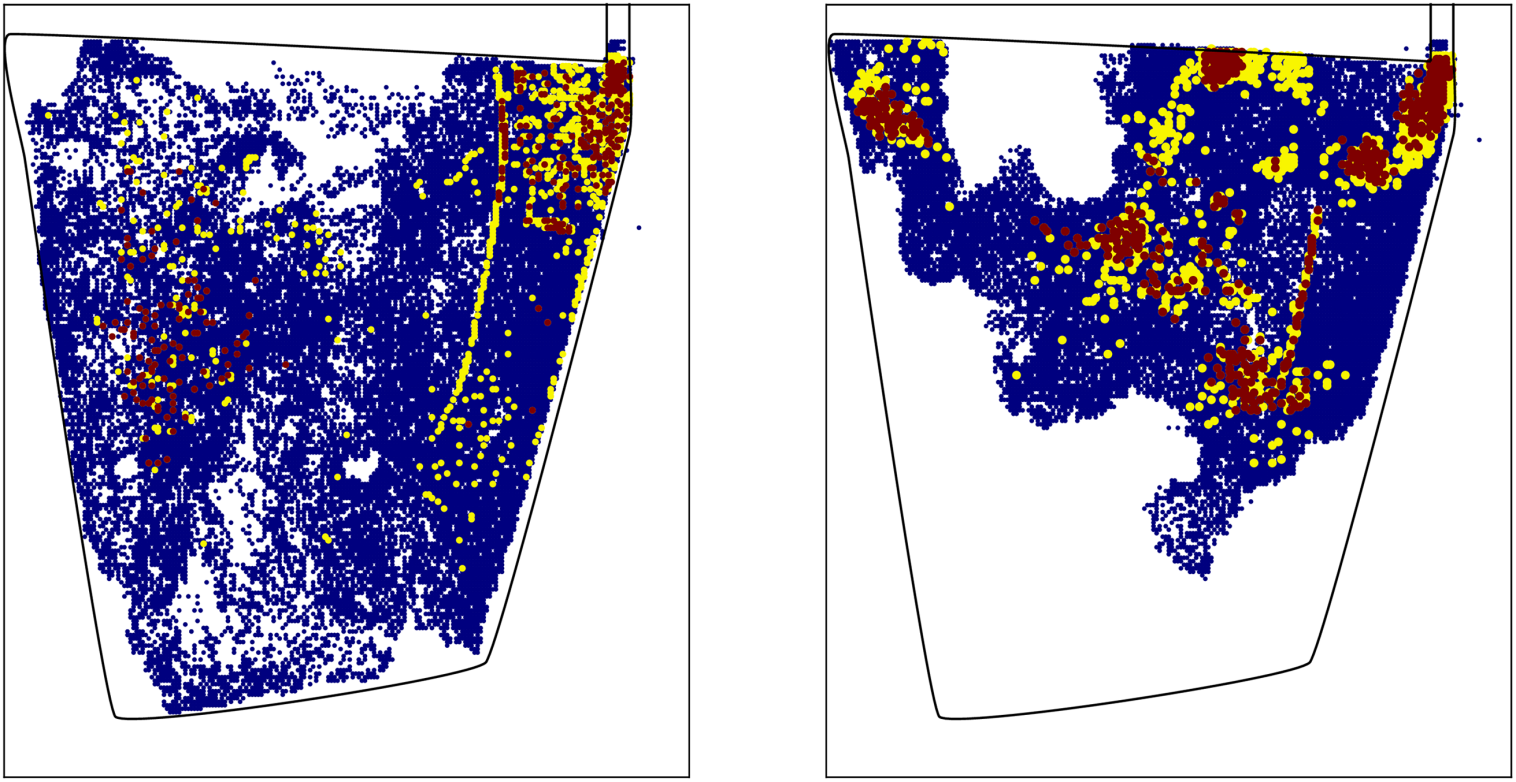
A heat map representation of two data sets used in the artificial mixing experiment. In each of the heat maps the data collection area was split into 45,576 equally sized rectangular cells. The number of observations that occurred in each of these cells was then enumerated. In both plots this enumeration was done using the data from the entire flock. Cells given in blue correspond to areas in which at least one observation was noted. Yellow and red cells correspond to areas where at least 0.01% and 0.05% of the observations occurred. This roughly corresponds, respectively, to 4.5 and 22.5 times the number of observations that would have been seen had the observations been uniformly spread over the rectangular cells. It can be observed that in both of the individual data sets the flock spends a disproportionately large amount of time in the top right section of the field. Properties such as this, which occur across the individual data sets, may go some way to accounting for the fact that the false-positive rate is slightly higher than expected in the artificial mixing experiment.

To check the performance of the significance test we calculate the connectivity levels both within and between the two sub-flocks. (The term connectivity level refers to the proportion of edges present in comparison to the total number of possible edges. For example, a connectivity level of one indicates that all possible edges are present.) The results are given in Fig 7. As expected, the level of connectivity within the two sub-flocks is far higher than between the two sub-flocks. The proportion of connections between the two flocks (i.e. the false-positive rate) was 4.9 ± 1.6%, which is slightly higher than expected. An explanation for this is that, even though we expect the behaviour of the two sub-flocks to be disparate, certain heterogeneous aspects of the data are causing a higher false-positive rate than expected. An illustration of this fact is given in Fig 8. It can be seen that the animals spend a disproportionately large amount of time around the entrance of the field, which is where the drinking trough was located. Given this heterogeneous aspect of the data, we applied the significance test to an irregular partition of the sample space, with all other aspects of the test remaining the same. In particular, we used algorithm 1 in Table 1 to construct a partition of the sample space such that roughly the same amount of observations fell into each of the subregions of the partition. The results from using this partition are given in Fig 7, with a false-positive rate of 2.6 ± 0.6%. This is significantly lower than the false-positive rate obtained when using a regular partition. This result shows the benefit of using irregular partitions of this form when the data sets is highly heterogeneous.

### Genuine Mixing Experiment

**Fig 9:**
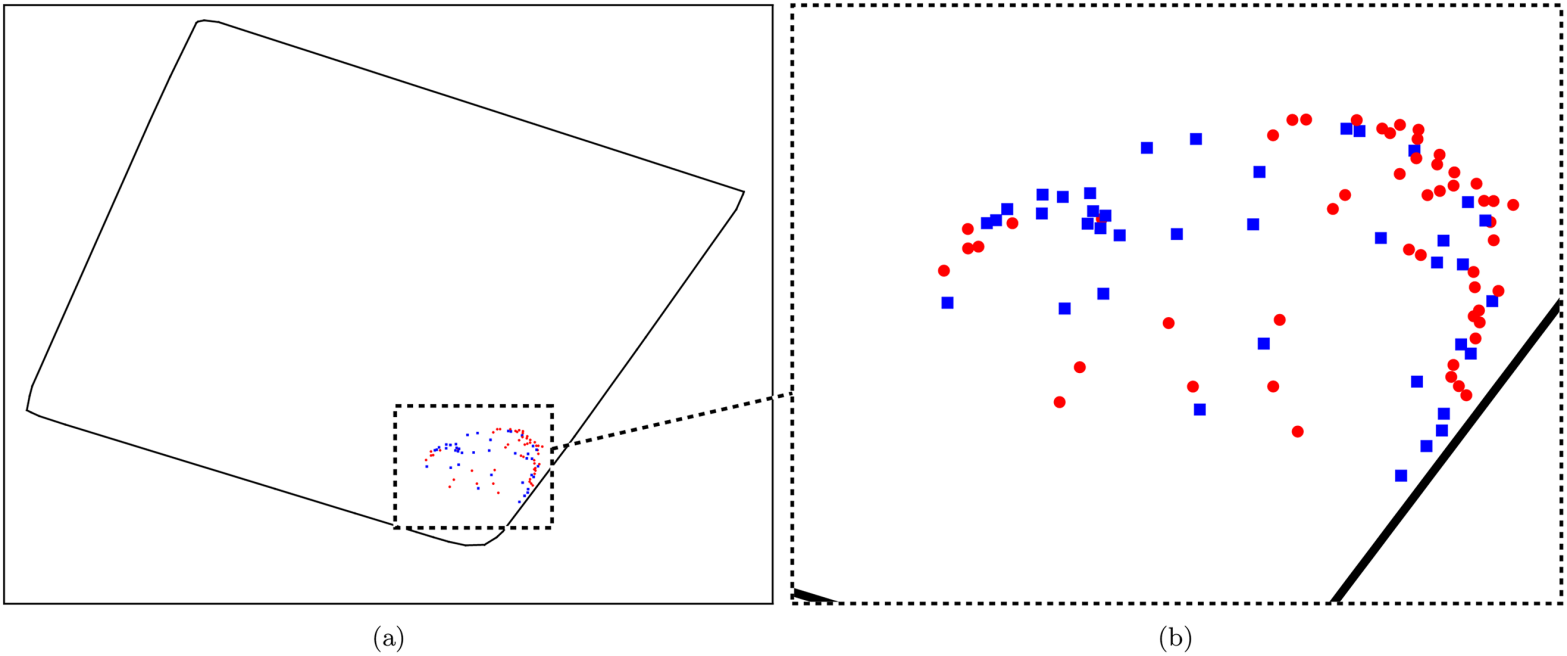
A snapshot of the movement patterns of the flock in the genuine mixing experiment. Members of one of sub-flock are depicted using red circles, whiles members of the other sub-flock are depicted using blue squares. (a) The position of the flock in relation to the field at an instant during the data collection period. The boundary of the field is denoted with a solid black line. (b) A close-up image of the flock during the same instant as in (a).

In the genuine experiment, we consider a data set that consists of ninety one individuals. In this data set the flock is formed of two sub-flocks of roughly equal size that are maintained as separate flocks for most of the year. The two sub-flocks have been merged into a single main flock once or twice a year over the past four years (on seven occasions in total) for 2–4 weeks on each occasion. Data used for this experiment were recorded immediately after mixing of the two sub-flocks. The aim of this genuine mixing experiment is the study of the social interactions during the mixing of the two sub-flocks. This data set allows us to determine the ability of the significance test to detect the mixing patterns exhibited between the two sub-flocks. We constructed a Matlab script that allowed us to examine the movement patterns of the flock, while the members of the two sub-flocks were highlighted in different colours. This script allowed us to examine how the two sub-flocks mixed over the course of the mixing experiment. Using this script it was clear that, while the two sub-flocks formed a single flock, the affiliations within the two sub-flocks were stronger than the affiliations between the two sub-flocks. A snapshot of this behaviour is given in Fig 9, which shows the location of the flock during an instant of the data collection period. It can be seen in Fig 9a that the two sub-flocks have formed a single flock, with the flock occupying a comparatively small proportion of the field. However, despite forming a single flock, it can be seen in 9b that the position of the animals within the flock is not independent of the sub-flock from which the animals originate. This gives a clear indication that the affiliations are stronger within the two sub-flocks than they are between them.

We consider mixing data over a continuous twenty two hour period, during which the animals were all in the field and were exhibiting their natural behaviour. We use the significance test to construct a weighted affiliation network of the flock during this period. We partition the sample space by first finding a rectangular region that bounds the field. We construct an irregular partition of this region, using algorithm 1 in Table 1, consisting of twenty five subregions. We take the median position of each individual over a five minute period as an observation. We consider a Markov model and use the data of the entire group to construct a single model. We consider a 0.5% level of significance in the test. We use the baseline approach to calculate the edge weights. The affiliation network generated by the significance test is given in Fig 5. (This representation of the affiliation network was generated using the spring layout of the NetworkX library [25], which implements the Fruchterman-Reingold force-directed algorithm [26].) A qualitative inspection of the affiliation network supports our visual inspection of the data, and suggests that associations are stronger within the two sub-flocks than between them. The mean non-zero edge weight within the two sub-flocks was 0.190 and 0.187 respectively, while the mean non-zero edge weight between the two sub-flocks was 0.076. The connectivity level within the two sub-flocks was 0.960 ans 0.988 respectively, and the connectivity level between the two sub-flocks was 0.719. To determine whether these results are significant we perform a permutation test for each of these six statistics of the affiliation network, i.e. the mean non-zero edge weight within the two sub-flocks and between the two sub-flocks, and the connectivity level within the two sub-flocks and between the two sub-flocks. Under the null hypothesis that sub-flock membership has no relation to the amount of association between animals in the flock, permuting the sub-flock membership labels of the animals will have no significant effect on the mean non-zero edge weight within the two sub-flocks or between the two sub-flocks. The same is true for the connectivity level within the two sub-flocks and between the two sub-flocks. We therefore assume this null hypothesis to be true, perform *n* ∈ ℕ permutations of the sub-flock membership labels, and calculate the six test statistics for each of these permutations. The null hypothesis is rejected at a given level of significance, *α* ∈ [0, 1], if the observed test statistic is higher/lower than *αn* of the test statistics obtained from the *n* permutations of the sub-flock membership labels. In all tests we used a value of *n* = 10,000, and used a significance level of *α* = 0.005. The null hypothesis was rejected in all six permutation tests, and we conclude that associations are stronger within the two sub-flocks than between them. We conclude that the significance test is correctly able to identify this structural aspect of the affiliation network for this group of animals.

Understanding social networks is challenging, not least because collecting the data manually is time consuming and requires trained observers. Determining social structure within even small groups of animals is difficult, and in large groups is nigh on impossible. No study of social behaviour within large groups of sheep has been conducted previously. This is understandable, because with conventional observation and recording, the problems with collecting data from flocks of sheep quickly become apparent. First, the flocking behaviour of sheep means that the distance between individuals is often small. Second, they move in a mob, so keeping track of an individual is difficult unless observed from above. Finally, even to the experienced observer, their lack of distinguishing features means that, once in a group, sheep are difficult—if not impossible—to tell apart. The consequence of this is that, whilst the emphasis of this paper lies in the methodology of establishing principled affiliation networks, this is the first detailed study that examines whether cohorts of sheep maintain social links when mixed. Using the analytical approach described in this paper, we are able to report that whilst the mixed flock we used was overtly cohesive, even to an experienced observer, social ties both exist and persist very strongly following mixing, emerging as spatial subgroupings within the flock.

A detailed conventional social network analysis of the flocks is clearly possible, given the ability to construct affiliation networks. However, it is outside the scope of this study. Whilst we believe this to be an important next step, the difficulties in validation and the general lack of large-group animal data mean that the interpretation of such an analysis is not straightforward and, as a result, this forms the subject of ongoing work.

## Summary

In this paper we have introduced a novel significance test for inferring the underlying affiliation network of a group from the movement patterns of its members. This was done by making the null hypothesis that the movements of the individuals are independent of each other, and then testing the significance of the evidence against this hypothesis. The test was constructed by first fitting a model of the individual movement patterns with respect to a finite partition of the data collection region, and then using this model to determine the significance of any similarities in observed movement patterns. We have empirically validated our approach on complex real-world data sets, consisting of the movement patterns of flocks of Merino sheep.

One of the main strengths of our approach in comparison to existing methods, such as the popular gambit of the group approach, is that is has a firm statistical grounding. The lack of any such grounding in the gambit of the group necessitates the setting of certain critical parameters in an ad-hoc manner, with these parameters typically set by hand using expert knowledge of the animals in the study. Using our real-world data sets, we have demonstrated the importance these parameters have in determining the affiliation network generated through the gambit of the group. The lack of a principled approach to setting the parameters of the gambit of the group can therefore be seen to be a serious drawback of the approach. By contrast, in our statistical approach there is a principled procedure both for specifying the parameters of the individual movement models, through maximum likelihood estimation, and also for determining the presence of an edge in the affiliation network, through our novel significance test. Whilst our approach does require the specification of a discretisation of the sample space, we have shown that the approach is robust to this specification. Furthermore, analogous significance tests in the statistics and machine learning literature have been shown to be statistically consistent when the granularity of the discretisation increases in line with the number of data points. Such consistency results should be obtainable for our approach. This remains a point for future research.
